# A case report of mixed left ventricular non-compaction/hypertrophic cardiomyopathy phenotype in a child

**DOI:** 10.1093/ehjcr/ytaf051

**Published:** 2025-02-14

**Authors:** Chiara Cirillo, Emanuele Monda, Santo Dellegrottaglie, Alessandra Scatteia, Giuseppe Limongelli

**Affiliations:** Department of Translational Medical Sciences, Inherited and Rare Cardiovascular Diseases, University of Campania ‘Luigi Vanvitelli’, Monaldi Hospital, Via Leonardo Bianchi, 80131 Naples, Italy; Department of Translational Medical Sciences, Inherited and Rare Cardiovascular Diseases, University of Campania ‘Luigi Vanvitelli’, Monaldi Hospital, Via Leonardo Bianchi, 80131 Naples, Italy; Advanced Cardiovascular Imaging Unit, Ospedale Medico-Chirurgico Accreditato Villa dei Fiori, Corso Italia, 110, 80018 Mugnano di Napoli, Naples, Italy; Advanced Cardiovascular Imaging Unit, Ospedale Medico-Chirurgico Accreditato Villa dei Fiori, Corso Italia, 110, 80018 Mugnano di Napoli, Naples, Italy; Department of Translational Medical Sciences, Inherited and Rare Cardiovascular Diseases, University of Campania ‘Luigi Vanvitelli’, Monaldi Hospital, Via Leonardo Bianchi, 80131 Naples, Italy; Institute of Cardiovascular Science, University College London, Gower St, London WC1E 6BT, UK

**Figure:**
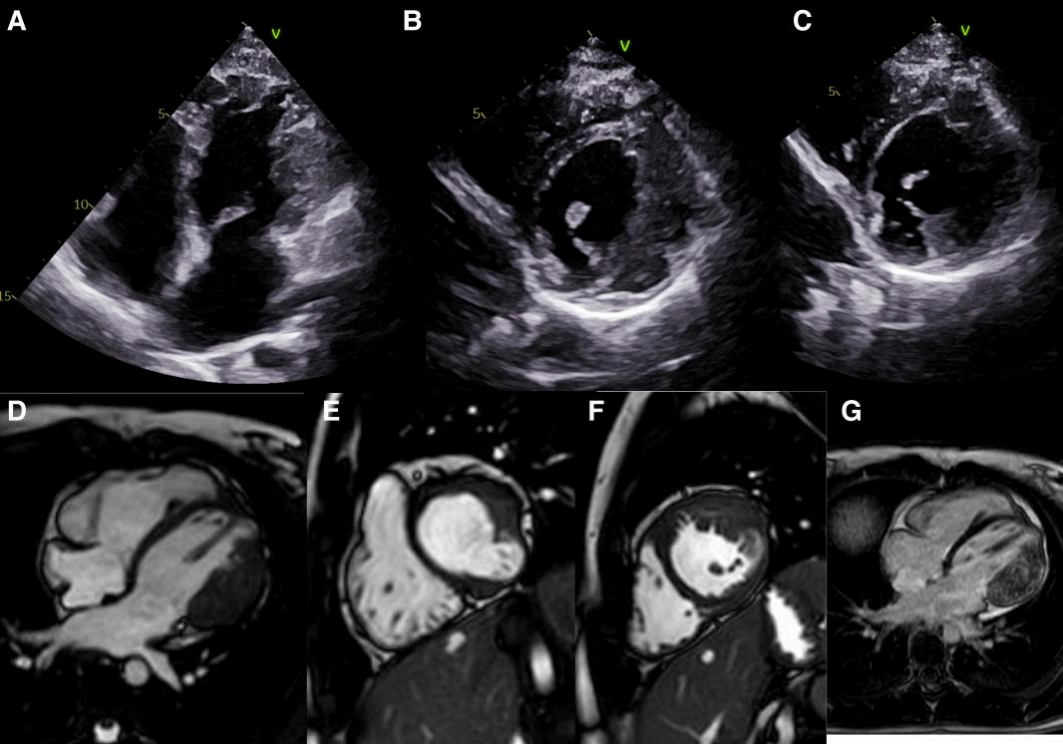


Hypertrophic cardiomyopathy (HCM) is characterized by left ventricular hypertrophy (LVH) not solely explained by abnormal loading conditions. Hypertrophic cardiomyopathy is rare in paediatric age and carries a relevant risk of mortality and morbidity in both infants and children.

We present the case of a 12-year-old girl who underwent surgical correction of subvalvular pulmonary stenosis at age 1. At age 11, she presented with a mixed cardiomyopathy phenotype with features of both HCM and left ventricular non-compaction (LVNC).

Her electrocardiogram showed sinus rhythm with incomplete right bundle branch block, and her echocardiogram showed asymmetric LVH of the lateral/inferolateral walls with hypertrabeculation and crypts (*Panels A* and *B*; Supplementary material online, *Videos S1* and *S2*). There was also evidence of a thinned-out area in the inferior wall in short axis (*Panel C*). No dynamic outflow obstruction or mid-wall gradient was detected on pulsed/continuous Doppler interrogation. There was no residual obstruction of the right ventricular outflow tract.

Cardiac magnetic resonance confirmed the presence of asymmetric LVH, particularly in the anterolateral wall with maximum wall thickness of 16 mm (*Panels D–F*; Supplementary material online, *Videos S3* and *S4*) with hypertrabeculation and crypts reaching LVNC criteria (NC/C > 2.3). Left ventricular systolic function was preserved with a left ventricular ejection fraction of 55%. There was an inferior wall aneurysm and late gadolinium enhancement imaging showed diffuse fibrosis of the hypertrophied areas.

A RASopathy was clinically and genetically excluded. A cardiomyopathy next-generation sequencing panel, containing syndromic and non-syndromic genes, was performed in the patient and her parents and showed a *de novo* pathogenic variant in *MYL2* gene (c.484G > A, p.Gly162Arg), known to be associated with HCM.

Her HCM Risk-Kids score was 6.3%. Therefore, she underwent a subcutaneous implantable cardioverter defibrillator implantation in primary prevention according to current ESC guidelines.

The *MYL2* p.Gly162Arg variant has been reported as a *de novo* occurrence in two individuals with HCM and in a third individual with LVH, reduced LVEF, and ST-segment abnormality (ncbi.nlm.nih.gov/clinvar/variation/132976). Furthermore, *in vitro* functional studies suggested that the p.Gly162Arg variant may impact protein function; however, these types of assays sometimes do not accurately represent biological function.

This is the first report of a mixed LVNC/HCM phenotype caused by a *MYL2* variant known to be pathogenic for HCM.

## Supplementary Material

ytaf051_Supplementary_Data

## Data Availability

No new data were generated or analysed in support of this research.

## Notes

**Consent:** The authors confirm that written consent for submission and publication of this case report, including images and associated text, has been obtained from the patient’s parents in line with COPE guidelines.

**Funding:** None declared.

